# Reply to Tarihci Cakmak et al. Comment on “Kang et al. The Effective Way of Botulinum Toxin Injection to Reduce Bite Force: Preliminary Study. *Toxins* 2025, *17*, 519”

**DOI:** 10.3390/toxins18040166

**Published:** 2026-03-30

**Authors:** Kun-Hwa Kang, Jae-Kwang Jung, Jin-Seok Byun, Ji Rak Kim

**Affiliations:** 1 Department of Oral Medicine, School of Dentistry, Kyungpook National University, Daegu 41940, Republic of Korea; 2 Craniofacial Nerve-Bone Network Research Center, Kyungpook National University, Daegu 41940, Republic of Korea

We thank the authors for their thoughtful comments [1] and the opportunity to clarify the scope and intent of our study [2].

The title of the present study refers specifically to an effective method for reducing bite force and does not intend to address the treatment of temporomandibular disorders as a whole or the overall therapeutic efficacy of botulinum toxin type A. Among the multiple mechanisms of action of botulinum toxin, this study focused exclusively on masticatory muscle paralysis and aimed to quantify the extent of bite force reduction and its duration over time. Accordingly, it was essential to control for other variables known to influence bite force in order to isolate the direct muscular effects of the injection.

If parameters such as pain, chewing efficiency, or patient-reported outcomes had been evaluated concurrently, changes in bite force could have been influenced by factors other than injection site-specific muscle paralysis, leading to conclusions driven by secondary clinical variables rather than the primary biomechanical effect. Although a reduction in bite force following injection into jaw-closing muscles is a predictable outcome, there were no prior studies providing quantitative data on the magnitude of this reduction or its temporal recovery pattern. In this context, we believe that the present study offers meaningful and novel information.

It is self-evident that the treatment of temporomandibular disorders ultimately aims at functional recovery and improvement in quality of life. However, achieving this goal requires a detailed and stepwise investigation of individual outcome components. From a clinical perspective, systematically examining specific parameters one at a time is an essential approach to building a solid evidence base for comprehensive patient-centered care.

As noted above, participants without temporomandibular pain or functional impairment were deliberately selected in order to control for variables that could influence bite force. We acknowledge that the inclusion of only male participants represents a clear limitation of the present study. During participant recruitment, asymptomatic individuals without pain were required, and in this process, male volunteers showed a higher willingness to participate than female volunteers.

In addition to this limitation, several other methodological constraints were present. For this reason, the title was revised to indicate that this work is a preliminary study. Furthermore, we explicitly stated in the Discussion and Conclusion sections that the findings should not be overinterpreted, and that the results should not be misconstrued as suggesting that injections into all three masticatory muscles are necessary or appropriate for all patients.

We clearly acknowledge as a limitation that injection treatment could not be performed in a double-blind manner in the present study. Implementing a double-blind design would have required administering saline injections to standardize injection sites in healthy adult participants. However, this approach could have induced iatrogenic pain, raising ethical concerns and making it difficult to obtain informed consent from asymptomatic volunteers. For these reasons, a double-blind design was not feasible in this study.

With regard to injections into the medial pterygoid muscle, we agree that ultrasound or electromyography guidance can improve injection accuracy. Nevertheless, when an extraoral approach via the submandibular region is used rather than an intraoral approach, the injection can be performed safely without guidance devices. In the present study, all injections were administered by the corresponding author, who is a board-certified specialist in oral medicine with more than 10 years of clinical experience in injection-based treatments.

We agree with the cited literature indicating that repeated or multi-muscle injections for the treatment of masseter hypertrophy may lead to reduced electromyographic activity and impaired muscle function without providing additional therapeutic or esthetic benefits. In this context, the present study was designed to explore injection strategies that could prolong the duration of bite force reduction with a single treatment session.

We do not suggest that the injection range should be routinely extended to include the medial pterygoid muscle in all patients. Rather, our findings indicate that medial pterygoid injection may be considered selectively, depending on individual clinical circumstances—for example, in cases where a reduction in bite force is desired but injection into the masseter muscle is not feasible, where maximal reduction in bite force is required, or where prolongation of the duration of bite force reduction is clinically necessary.

At present, commercially available devices capable of measuring absolute bite force are limited, and to our knowledge, Dental Prescale and Innobyte are among the few systems currently in use. Because numerous previous studies have reported bite force measurements obtained using the Dental Prescale system, we selected the same device to ensure methodological consistency and comparability with existing literature.

The operational procedure of the Dental Prescale system is relatively straightforward and can be sufficiently standardized through several practice sessions. The measurement protocol, including the conditions under which bite force was assessed, was described in detail in the Methods section.

For asymptomatic healthy volunteers, observation of post-injection changes in bite force did not provide any direct personal benefit. Consequently, attending scheduled follow-up visits over a predefined study period may have imposed a burden on participants. The higher dropout rate observed in the multi-muscle injection group was primarily attributable to the longer follow-up duration required for this group compared with the other groups.

Although an intention-to-treat analysis can reduce the risk of bias associated with differential dropout, its application in the present study would have required imputation of missing longitudinal data. Given the small sample size, the exploratory nature of this preliminary study, and the focus on quantifying the direct biomechanical effects of muscle-specific botulinum toxin injections, such imputation could have increased statistical uncertainty, diluted effect estimates, and potentially distorted the true temporal pattern of bite force changes. For these reasons, we considered a per-protocol analysis to be more appropriate for addressing the specific aims of this study, while acknowledging the increased risk of bias as an inherent limitation.

We fully agree with the points raised in this regard. We would like to emphasize that the findings of the present study should not be interpreted as indicating that multi-muscle injections provide superior therapeutic efficacy. Rather, our results should be understood as demonstrating that multi-muscle injections may be helpful in reducing bite force, without implying enhanced overall treatment outcomes.
